# Arsenic trioxide reduces chemo-resistance to 5-fluorouracil and cisplatin in HBx-HepG2 cells via complex mechanisms

**DOI:** 10.1186/s12935-015-0269-y

**Published:** 2015-12-12

**Authors:** Guifang Yu, Xuezhu Chen, Shudi Chen, Weipeng Ye, Kailian Hou, Min Liang

**Affiliations:** Department of Oncology, The Fifth Affiliated Hospital of Guangzhou Medical University, No. 621, Gangwan Road, Huangpu District, Guangzhou, 510700 China

**Keywords:** HBV, HCC, HBx-HepG2, 5-FU, Cisplatin, Chemo-resistance, HIF-1α, P-gp, MRP1, LRP

## Abstract

**Background:**

Multidrug resistance is one of the major reasons chemotherapy-based treatments failed in hepatitis B virus (HBV) related hepatocellular carcinoma (HCC). Hypoxia is generally associated with tumor chemo-resistance. The aim of the study was to investigate the effect of Arsenic trioxide (As_2_O_3_) on the hypoxia-induced chemo-resistance to 5-FU or cisplatin and explored its underlying mechanism in the HBx-HepG2 cells.

**Methods:**

MTT assay was used to examine the cell viability. Mitochondrial membrane potential (MMP) and cell cycle was examined by flow cytometry. qRT-PCR was employed to observe the mRNA expression level; and western blot assay was used to determine the protein expression level.

**Results:**

Our results showed that transfection of HBx plasmid established the HBx-HepG2 cells expressing HBx, and the expression of HBx was confirmed by qRT-PCR and western blot. Exposure of HBx-HepG2 cells to hypoxia (5 % O_2_, 3 % O_2_, 1 % O_2_) for 48 h increased the chemo-resistance to 5-fluorouracil (5-FU) (50–1600 µM) and cisplatin (25–800 µM), reduced MMP, and caused the cell cycle arrest at G_0_/G_1_ phase in a concentration-dependent manner. Hypoxia also concentration-dependently (5 % O_2_, 3 % O_2_, 1 % O_2_) reduced mRNA expression level of P-glycoprotein (P-gp), multidrug resistance protein (MRP1), lung resistance protein (LRP), and decreased the protein expression level of hypoxia-inducible factor-1α (HIF-1α), P-gp MRP1, and LRP. Following pretreatment with As_2_O_3_ at a non-cytotoxic concentration re-sensitized the hypoxia (1 % O_2_)-induced chemo-resistance to 5-FU and cisplatin in HBx-HepG2 cells. As_2_O_3_ pretreatment also prevented MMP reduction and G_0_/G_1_ arrest induced by hypoxia. Meanwhile, As_2_O_3_ antagonized increase of HIF-1α protein induced by hypoxia, and it also suppresses the increase in expression levels of P-gp, MRP1, and LRP mRNA and proteins. In addition, As_2_O_3_ in combination with 5-FU treatment caused up-regulation of DR5, caspase 3, caspase 8, and caspase 9, and down-regulation of BCL-2, but had no effect of DR4.

**Conclusions:**

Our results may suggest that As_2_O_3_ re-sensitizes hypoxia-induced chemo-resistance in HBx-HepG2 via complex pathways, and As_2_O_3_ may be a potential agent that given in combination with other anti-drugs for the treatment of HBV related HCC, which is resistant to chemotherapy.

## Background

Hepatitis-B-virus (HBV)-related hepatocellular carcinoma (HCC) is among the most common cancers in the world and is also the most frequent cause of death from this malignancy [1]. Although the precise mechanism underlying the development of HCC is unclear, several studies have shown that hepatitis virus X protein (HBx) plays a key role in the pathogenesis of HCC [2, 3]. HBx demonstrates its oncogenic potential in transgenic model [4]. In addition, HBx affects both mitochondria/caspase family and the cell-cycle check proteins [5–7].

HCC is characterized by a high degree of multidrug resistance (MDR) [8]. In clinic, it has been found that part of HCC patients exhibited MDR even during the course of initial chemotherapy instead of after repeated chemotherapy, which suggests that MDR might be the nature of some HCC. However, the detailed molecular mechanisms of MDR are largely unknown, and studies have shown that chemo-resistant solid tumors depend on the actions of drug transporter proteins, including P-glycoprotein (P-gp), multidrug resistance protein (MRP1), lung resistance protein (LRP) and others, which export actively most anticancer drugs to decrease intracellular drug accumulation from tumor cells [9, 10].

The microenvironment around rapidly growing HCC is associated with increase oxygen consumption and relatively diminished blood supply, which results in prominent hypoxia of focal areas [11]. Hypoxia has been shown to enhanced tumor progression in HCC [12]. Hypoxia-inducible factor-1 (HIF-1) has been shown to an important mediator of hypoxia-regulated gene expression, and it can activate the transcription of genes that are involved in key aspects of cancer biology, including cell viability, invasion, metastasis, and angiogenesis [13–15].

Arsenic trioxide (As_2_O_3_) has shown promising results in the treatment of many hematopoietic malignancies. In the 1970s, Chinese physicians began to use As_2_O_3_ to treat actue promyelocytic leukemia (APL) with great success [16, 17]. Although the detailed molecular mechanisms of arsenic trioxide are not clarified, results in various studies have showed that As_2_O_3_ involves in induction of apoptosis, partial cellular differentiation, degradation of specific APL fusion transcripts, anti-proliferation, generation of reactive oxygen species [18, 19]. Recent studies also demonstrated that As_2_O_3_ reduces drug resistance to adriamycin in leukemic K562/A02 cells via multiple mechanisms [20], and arsenic trioxide eliminates the differential chemo-resistance in B cell lymphoma cells via the overexpression of p28 [21]. Another study showed that As_2_O_3_ re-sensitized cells to 5-fluorouracil (5-FU) in 5-FU-resistant colorectal cancer cell line via suppression of thymidylate synthase [22]. In the HCC, As_2_O_3_ potentiates the anti-cancer activities of sorafenib via inhibiting Akt activation [23]. Studies also demonstrated that As_2_O_3_ reduced chemo-resistance via inducing apoptosis in cancer cell lines [24], and the apoptosis induced by As_2_O= has been suggested to be mediated by tumor necrosis factor-related apoptosis-inducing ligand (TRAIL) [25], which is important in regulating the apoptotic signaling pathways [26–29]. For instance, As_2_O_3_-mediated growth inhibition of myeloma cells is associated with an extrinsic or intrinsic signaling pathway through activation of TRAIL or TRAIL receptor two [30] and another line of study demonstrated that As_2_O_3_ sensitizes human glioma cells to TRAIL-induced apoptosis via CCAAT/enhancer-binding protein homologous protein-dependent DR5 up-regulation [31]. However, whether As_2_O_3_ could re-sensitize the chemo-resistant HBV-related HCC to anti-cancer drugs is still unknown.

In the present study, we first established the stable HepG2 cell lines expressing HBx. Then, we determined the hypoxia conditions, which induce chemo-resistance to 5-FU and cisplatin by using MTT assay. The effects of different hypoxia conditions on MMP and cell cycle were also examined by using flow cytometry. qRT-PCR and western blot assay were used determine the effect of hypoxia treatment on the expression levels of HIF-1α, P-gp, MRP1, and LRP. We also investigated if pretreatment of As_2_O_3_ could re-sensitize the hypoxia-induced chemo-resistance in HBx-HepG cells, and also examined its effect on MMP, cell cycle as well as the expression levels of HIF-1α, P-gp, MRP1, and LRP. Finally, we examined the effect of As_2_O_3_ in combination with 5-FU treatment on apoptotic signaling pathway.

## Methods

### Cell culture

The cells of HepG2 (American Type Culture Collection, USA) and HepG2.2.15 (constitutively replicate HBV) (Shanghai Second Military Medical University), were cultured in Dulbecco’s modified Eagle medium (DMEM) with 10 % fetal bovine serum (Life Technologies Inc, Gaithersburg, MD, USA). Cell were incubated at 37 °C in a humidified atmosphere with 5 % CO_2_.

### Generation of stable HepG2 cell lines expressing HBx

The HBV X gene was amplified from plasmid pIERES2-EGFP-HBV by PCR. The purified HBx gene fragment was inserted into a lentivirus vector (pZac2.1). HepG2 cells were then transfected with the packaged recombinant lentivirus, and resistant cell clones were selected with puromycin. The expression of HBx was examined using qRT-PCR and Western blot.

### Establishment of hypoxia model and drug treatment

Cells were cultured under normoxia condition for 24 h and subsequently were culture under normoxic (21 % O_2_, 5 % CO_2_, 74 % N_2_) or hypoxic condition (5 % O_2_, 5 % CO_2_, 90 % N_2_; 3 % O_2_, 5 % CO_2_, 92 % N_2_; or 1 % O_2_, 5 % CO_2_, 94 % N_2_) for 48 h in the tris-air incubator (NU4950, NUAIRE company, USA).

### MTT assay

Cells were plated at 5000 cells per well in 96-well plates. After culture for 24 h under normoxic conditions, cells were exposed to normoxic (21 % O_2_) or hypoxic (5 % O_2_, 3 % O_2_, or 1 % O_2_) conditions for 48 h. Cells were then treated with relevant doses of 5-FU (0, 50, 100, 200, 400, 800, 1600 μM) and cisplatin (0, 25, 50, 100, 200, 400, 800 μM) for 24 h. In another set of experiment, 48 h after hypoxia treatment, cells were pretreated with As_2_O_3_ for 24 h, and then treated with relevant doses of 5-FU (0, 50, 100, 200, 400, 800, 1600 μM) and cisplatin (0, 25, 50, 100, 200, 400, 800 μM) for 24 h. Subsequently, cells were incubated for 4 h in the presence of the MTT reagent and then lysed with acidified isopropanol. Absorbance was measured at 570 nm. Cell survival rate (cell viability) was calculated as the absorbance value of the test group/absorbance value of the control group X 100 %.

### RNA extraction, reverse transcription PCR and qRT-PCR analysis

Forty-eight hour after hypoxia (5 % O_2_, 3 % O_2_ or 1 % O_2_) treatment, or 48 h after hypoxia (1 % O_2_) treatment followed by As_2_O_3_ treatment for 24 h, total RNA fractions were isolated from HBx-HepG2 cells and HepG2.2.15 cells using TRIzol reagent (Invitrogen, CA, USA). qRT-PCR was performed with SYBR Premix Ex Taq (TaKaRa). GAPDH was used as an internal control for mRNA. The relative quantitative analysis of data was performed using 7000 system SDS software v1.2.3 (Applied Biosystems). The relative quantitation of target gene expression was obtained using the comparative ΔΔCT method.

### Flow cytometry

Forty-eight hour after hypoxia (5 % O_2_, 3 % O_2_ or 1 % O_2_) treatment, or 48 h after hypoxia (1 % O_2_) treatment followed by As_2_O_3_ treatment for 24 h, cells were subject to cell cycle and MMP analysis.

For the analysis of MMP, the cells were harvested and washed with ice cold PBS twice by centrifugation at 1000 rpm for 10 min, and then 0.5 ml PBS contained 10 µg/ml Rho-123 was added to the cells. The tubes were vortexed gently and incubated at 37 °C in the dark for 30 min, then were washed with ice-cold PBS twice by centrifugation at 1000 rpm for 10 min. 0.5 ml PBS was added to the cells. Flow cytometric analysis was carried out by FACSCalibur flow cytometer (BD Biosciences, San Jose, CA, USA).

For cell cycle analysis, cells were fixed with 75 % ethanol at 4 °C overnight and washed with cold PBS and treated with RNaseI, followed by a 30 min staining with propidium iodide in dark. Cell cycle distributors were analyzed by a FACSCalibur flow cytometer (BD Biosciences, San Jose, CA, USA).

### Western blot analysis

Forty-eight hour after hypoxia (5 % O_2_, 3 % O_2_ or 1 % O_2_) treatment, or 48 h after hypoxia (1 % O_2_) treatment followed by As_2_O_3_ treatment for 24 h, and in the apoptosis study, cells were further treated with 5-FU for another 24 h, cells were subject to western blot assay. Proteins were extracted from whole cell lysates and separated by sodium dodecyl sulfate–polyacrylamide gel electrophoresis, then transferred to a polyvinylidene fluoride (PVDF) membrane. The following primary antibodies were used: mouse-anti-HBx (1:1000; Abcam, Cambridge, MA, USA); rabbit anti-HIF-1α (1:1000; Abcam, Cambridge, MA, USA); rabbit anti-P-gp (1:500; Abcam, Cambridge, MA, USA); rabbit-anti-MRP1 (1:1500; Abcam, Cambridge, MA, USA); rabbit-anti-LRP (1:500; Santa Cruz Biotechnology, Dallas, TX, USA); rabbit-anti-death receptor 4 (DR4) (1:500; Abcam, Cambridge, USA); rabbit-anti-death receptor 5 (DR5) (1:1000; Abcam, Cambridge, MA, USA); rabbit-anti-BCL-2 (1:1500; Abcam, Cambridge, MA, USA); rabbit-anti-caspase-3 (1:300, Santa Cruz Biotechnology, Dallas, Texas, USA); rabbit-anti-caspase-8 (1:2000, Santa Cruz Biotechnology, Dallas, Texas, USA); rabbit-anti-caspase-9 (1:1000, Abcam, Cambridge, MA, USA); and mouse anti-β-actin (1: 5000; Abcam, Cambridge, MA, USA). Membranes were then incubated with the horseradish peroxidase-conjugated secondary anti-bodies (1:2000; Abcam, Cambridge, MA, USA). The membranes were exposed and with the Image J program (Bio-Rad).

### Statistical analysis

All statistical analysis was carried out using GraphPad Prism version 6 (GraphPad Prism version 6.0, Inc., San Diego, CA, USA). The differences among groups were analyzed by one-way ANOVA followed by Bonferroni’s multiple comparison tests or t-test, as appropriate. All data are expressed as mean ± s.e.m. Differences were considered significant when *P* < 0.05.

## Results

### Generation of HBx-HepG2 cell lines

**Fig. 1:**
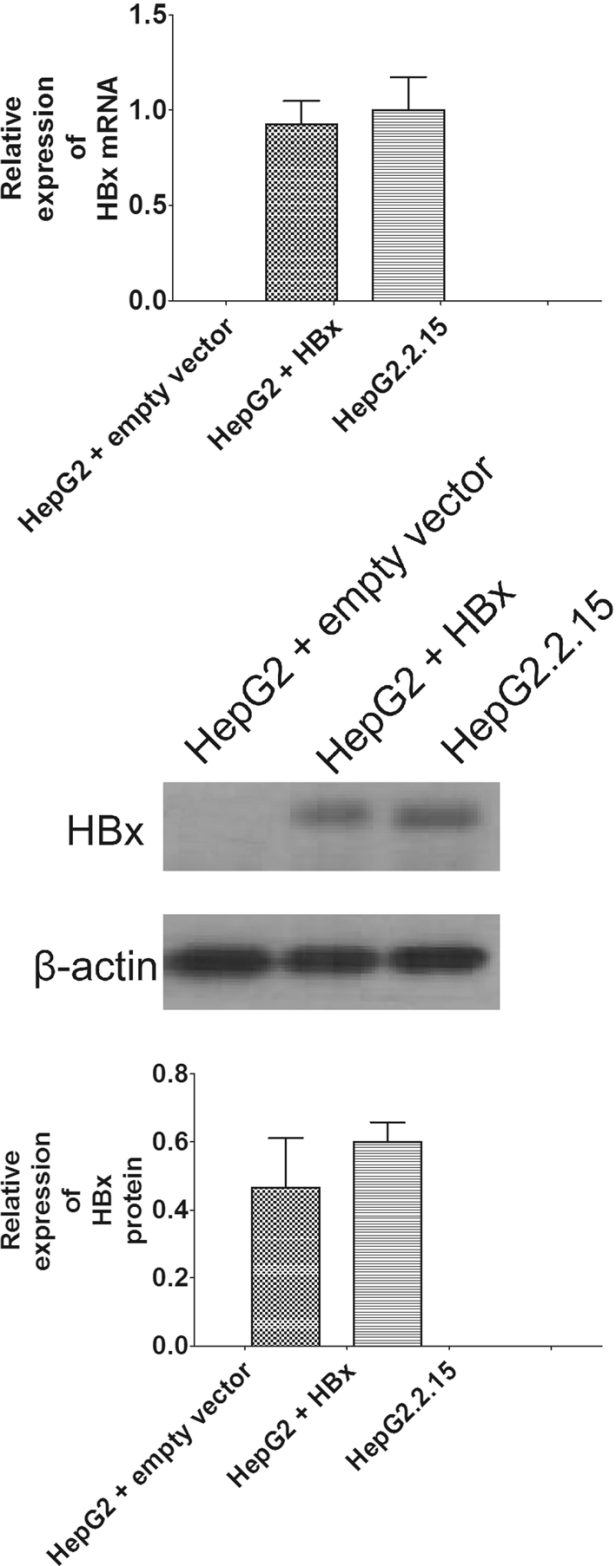
Establishment of HepG2-HBx cell lines. qRT-PCR and western blot were used to evaluate the expression levels of HBx mRNA and protein in empty vector-transfected HepG2 cells, HBx-transfected HepG2 cells and HepG2.2.15 cells. All data were expressed as mean ± s.e.m., n = 4

After transfecting the HepG2 cells with HBx plasmid or empty vector, qRT-PCR and western blot results showed that HBx mRNA and protein were expressed in the HBx-transfected HepG2 cell lines, and the expression levels of HBx mRNA and protein were similar to that in positive control, HepG2.2.15 cells, which stably expressed the whole HBV genome; no HBx expression were found in the HepG2 cells transfected with empty vector (Fig. 1).

### Effect of hypoxia on chemo-sensitivity

The chemo-sensitivity of the HBx-HepG2 cells to 5-FU (50–1600 μM) and cisplatin (25–800 μM) was evaluated under normoxic or hypoxic conditions using the MTT assay. 5-FU (50–1600 μM) and cisplatin (25–800 μM) dose-dependently reduced the cell viability under normoxic condition (21 % O_2_).

**Fig. 2:**
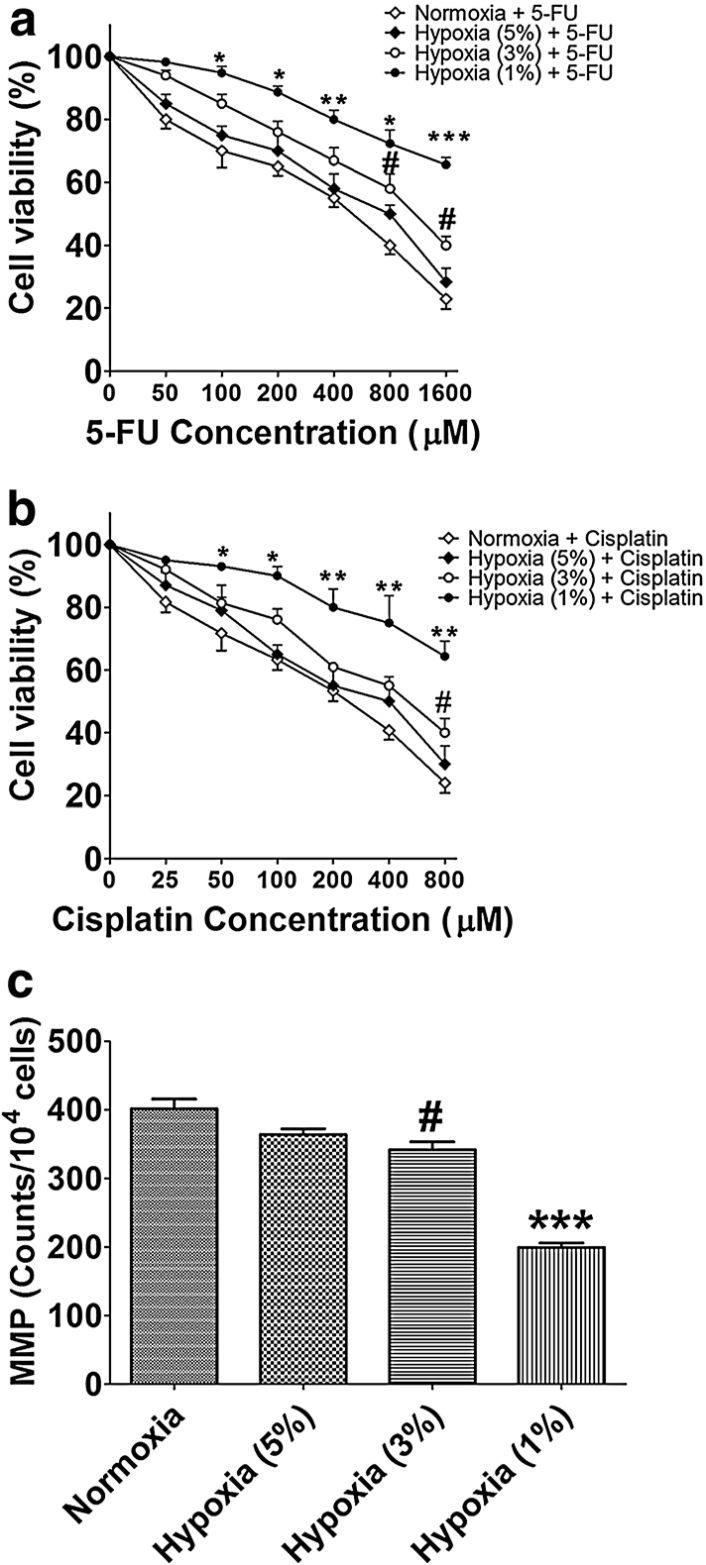
Effect of hypoxia on the cell viability of HBX-HepG2 cells treated with chemotherapeutic agents and on the mitochondrial membrane potential (MMP) in HBX-HepG2 cells. MTT assay was conducted on HBX-HepG2 cells pre-incubated for 48 h in normoxic (21 % O_2_) or hypoxic (5 % O_2_, 3 % O_2_, or 1 % O_2_) conditions and subsequently treated with **a** 5-FU (50–1600 µM) or **b** Cisplatin (25–800 µM); **c** flow-cytometry was conducted on HBx-HepG2 cells pre-incubated for 48 h in normoxic (21 % O_2_) or hypoxic (5 % O_2_, 3 % O_2_, or 1 % O_2_) conditions to measure the MMP. All data were expressed as mean ± s.e.m., n = 4; significant differences between hypoxia (1 % O_2_) group and normoxia group were indicated as *P < 0.05, **P < 0.01, ***P < 0.001; significantly differences between hypoxia (3 % O_2_) group and normoxia group were indicated as ^#^ *P* < 0.05 (One-way ANOVA followed by Bonferroni’s multiple comparison tests)

Exposure of HBx-HepG2 cells to hypoxia (5 % O_2_) for 48 h did not significantly affect the chemo-sensitivity to 5-FU and cisplatin (*P* > 0.05; n = 4, Fig. 2a, b); Exposure of HBx-HepG2 cells to hypoxia (3 % O_2_) for 48 h significantly increased the chemo-resistance to 5-FU and cisplatin at dose of 800- 1600 μM and 800 μM, respectively, (*P* < 0.05; n = 4, Fig. 2a, b); Exposure of HBx-HepG2 cells to hypoxia (1 % O_2_) for 48 h also significantly increased the chemo-resistance to 5-FU and cisplatin with much larger dose ranges (100–1600 μM for 5-FU; 50–800 μM for cisplatin) (*P* < 0.05; n = 4, Fig. 2a, b).

### Effect of hypoxia on MMP

Mitochondria involve in the process of apoptosis and necrosis in different ways and the MMP reflects the function of mitochondria. To examine the effect of hypoxia on MMP, we assessed the changes of MMP using rhodamine 123 (Rho-123). Flow cytometry showed that MMP was not affected in HBx-HepG2 cells exposed to hypoxia (5 % O_2_) for 48 h compared with the normoxia (21 % O_2_) group (*P* > 0.05; n = 4, Fig. 2c). Exposure of hypoxia (3 % O_2_ or 1 % O_2_) for 48 h significantly decreased the MMP in HBx-HepG2 cells compared with normoxia (21 % O_2_) group, and exposure of hypoxia (1 % O_2_) for 48 h showed about 50 % reduction of MMP compared with normoxia (21 % O_2_) group (*P* < 0.05; n = 4, Fig. 2c).

### Effect of hypoxia on cell cycle

The effect of different hypoxic conditions on cell cycle of HBx- HepG2 were analyzed by flow cytometry. Our results showed that exposure of HBx-HepG2 cells to hypoxia (5 % O_2_) for 48 h did not significantly affect the cell cycle (Table 1); exposure of HBx-HepG2 cells to hypoxia (3 % O_2_) for 48 h significantly increased percentage of G_0_/G_1_ phase compared with normoxia (21 % O_2_) group (63.7 vs. 60.2 %; Table 1); exposure of HBx-HepG2 cells to hypoxia (1 % O_2_) for 48 h significantly increased percentage of G_0_/G_1_ phase compared with normoxia (21 %, O_2_) group (67.9 vs. 60.2 %; Table 1), also with a concomitant decrease in the percentage of cells in the S phase (11.8 vs. 20.3 %; Table 1) and G_2_/M phase (7.0 vs. 14.6 %; Table 1).

**Table 1:** Effect of Hypoxia on the cell cycle of HBX-HepG2 cells

	Sub G_1_	G_0_/G_1_	S	G_2_/M
Normoxia	1.1 ± 0.4	60.2 ± 9.2	20.3 ± 3.7	14.6 ± 4.3
Hypoxia (5 %)	1.3 ± 1.1	61.5 ± 10.3	19.5 ± 6.8	14.5 ± 2.9
Hypoxia (3 %)	5.5 ± 3.3	63.7 ± 8.7^#^	17.5 ± 6.5	13.2 ± 2.3
Hypoxia (1 %)	12.9 ± 6.6	67.9 ± 11.2**	11.8 ± 4.9*	7.0 ± 5.5*

All data were expressed as mean ± s.e.m., n = 4; significant differences between hypoxia (1% O_2_) group and normoxia (21% O_2_) group were indicated as * *P* < 0.05, ** *P *< 0.01; significant differences between hypoxia (3% O_2_) group and normoxia (21% O_2_) group were indicated as ^#^ *P* < 0.05 (One-way ANOVA followed by Bonferroni’s multiple comparison tests)

### Effect of hypoxia on expression of MDR related genes and proteins

**Fig. 3:**
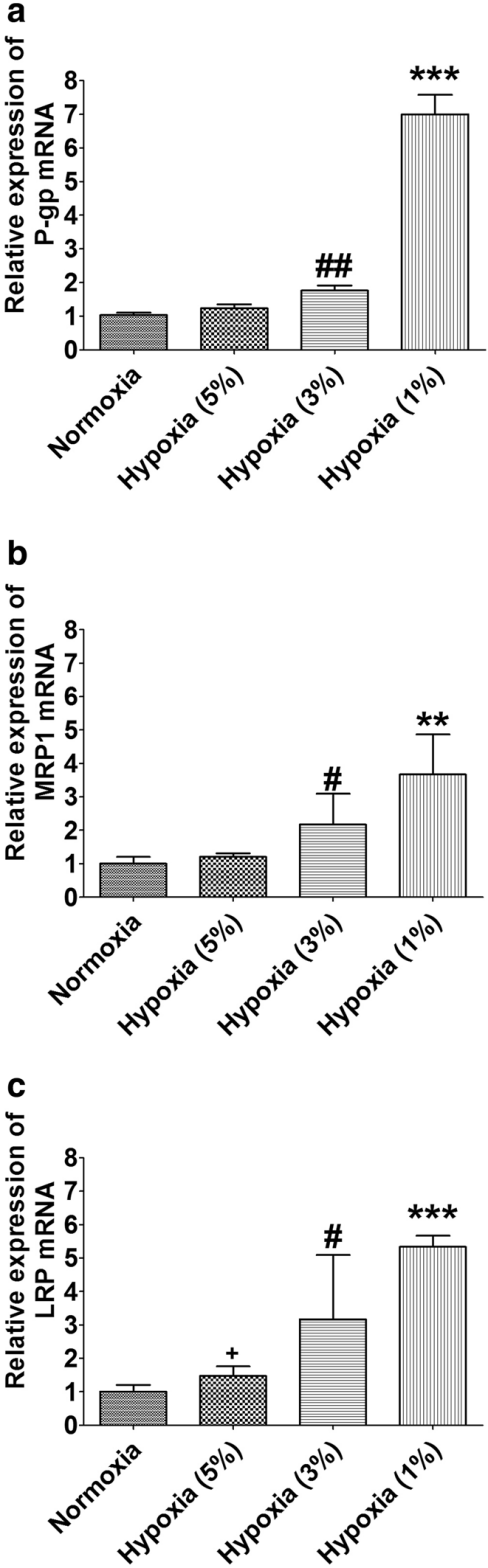
Effect of hypoxia on expression levels of P-gp, MRP1 and LRP mRNA inHBX-HepG2 cells. qRT-PCR assay was conducted on HBx-HepG2 cells pre-incubated for 48 h in normoxic (21 % O_2_) or hypoxic (5 % O_2_, 3 % O_2_, or 1 % O_2_) to measure the expression levels of **a** P-gp, **b** MRP1 and **c** LRP mRNA. All data were expressed as mean ± s.e.m., n = 4; significant differences between hypoxia (1 % O_2_) group and normoxia group were indicated as **P < 0.01, ***P < 0.001; significant differences between hypoxia (3 % O_2_) group and normoxia group were indicated as ^#^ *P* < 0.05; ^##^ *P* < 0.01 (One-way ANOVA followed by Bonferroni’s multiple comparison tests)

qTR-PCR was used to determine the expression of P-gp, MRP1, and LRP mRNA in HBx-HepG2 cells pre-incubated in normoxic (21 % O_2_) or hypoxia (5 % O_2_, 3 % O_2_, or 1 % O_2_) conditions for 48 h. Hypoxia exposure concentration-dependently (from 5 to 1 % O_2_) increased the expression levels of P-gp, MRP1, and LRP mRNA in HBx-HepG2 cells (*P* < 0.05;n = 4, Fig. 3) with hypoxia(1 % O_2_) causing about 6.5 fold, 3.5 fold and 5.0 fold increase of P-gp, MRP1, and LRP mRNA (*P* < 0.05; n = 4, Fig. 3).

**Fig. 4:**
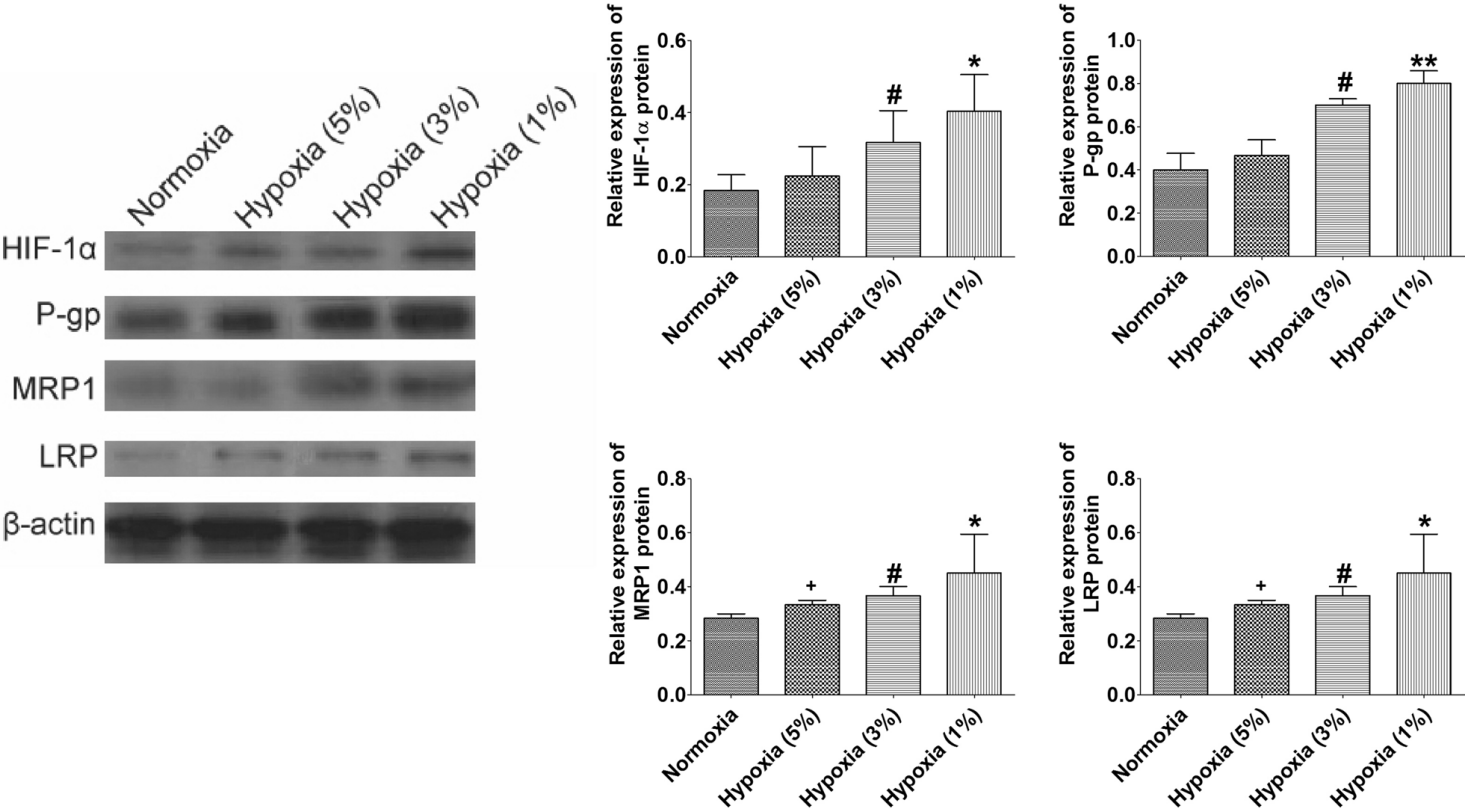
Effect of hypoxia on expression levels of HIF-1α, P-gp, MRP1 and LRP protein in HBx-HepG2 cells. Western blot assay was conducted on HBX-HepG2 cells pre-incubated for 48 h in normoxic (21 % O_2_) or hypoxic (5 % O_2_, 3 % O_2_, or 1 % O_2_) conditions to measure the expression levels of HIF-1α, P-gp, MRP1 and LRP protein, band densities were normalized to β-actin. All data were expressed as mean ± s.e.m., n = 4; significant differences between hypoxia (1 % O_2_) group and normoxia group were indicated as *P < 0.05, **P < 0.01; significantly differences between hypoxia (3 % O_2_) group and normoxia group were indicated as ^#^ *P* < 0.05 (One-way ANOVA followed by Bonferroni’s multiple comparison tests)

We further used western blot assay to examine the effect of hypoxia on the expression of HIF-1 α, P-gp, MRP1, and LRP protein in HBx-HepG2 cells. The western blot results showed that hypoxia also concentration-dependently (from 5 to 1 % O_2_) increased the expression levels of P-gp, MRP1, and LRP protein in HBx-HepG2 cells (*P* < 0.05; n = 4, Fig. 4) with hypoxia (1 % O_2_) having the most profound effect on the increased expression of P-gp, MRP1, and LRP protein (P < 0.05; n = 4, Fig. 4).

### As_2_O_3_ re-sensitized hypoxia-induced resistance to 5-FU and cisplatin in HBx-HepG2 cells

**Fig. 5:**
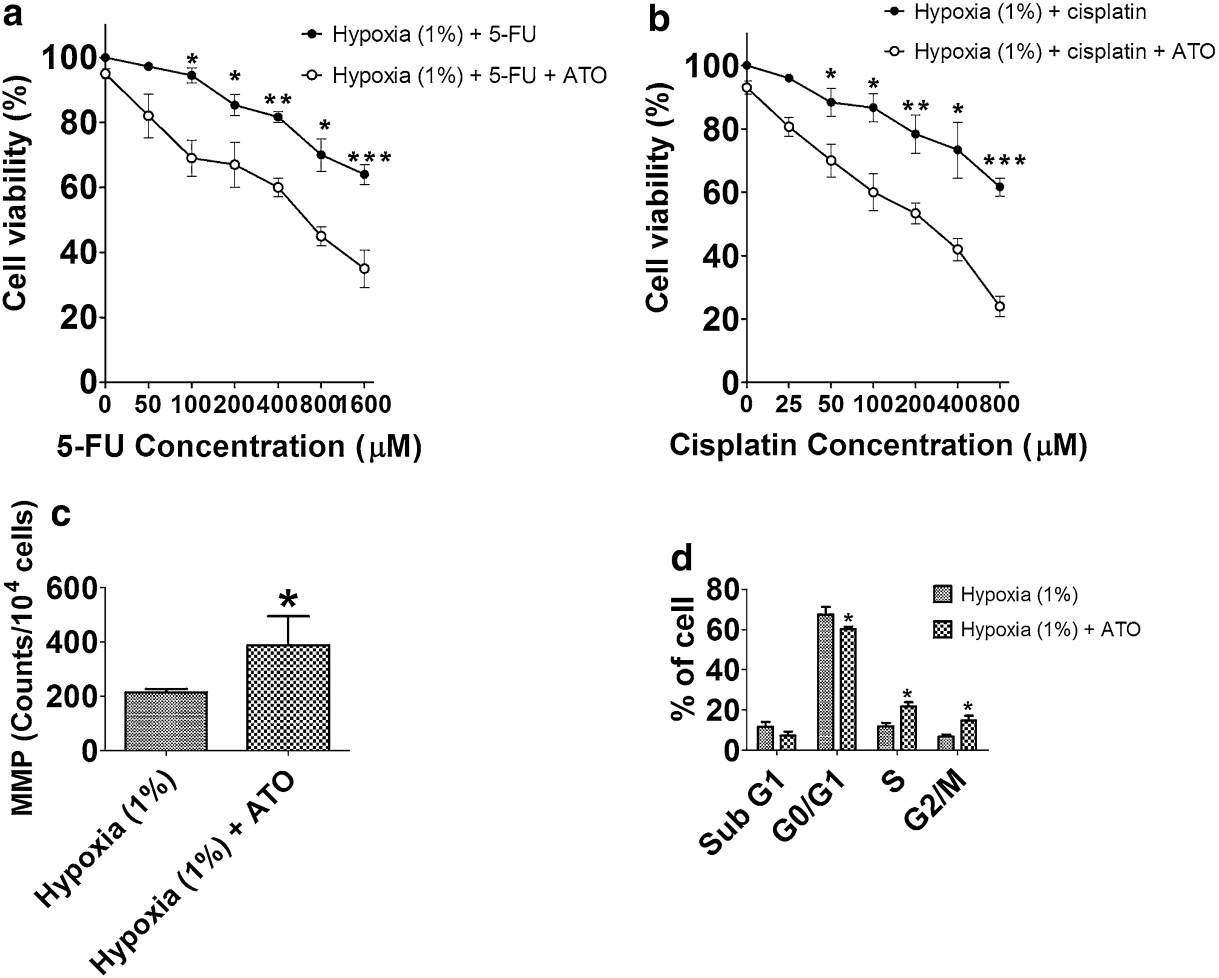
Effect of As_2_O_3_ (1 µM) on chemo-sensitivity, MMP, and cell cycle of HBx-HepG2 cells pretreated with hypoxia (1 % O_2_). MTT assay was used to evaluate the effect of As_2_O_3_ on chemo-sensitivity to **a** 5-FU (50–1600 µM) or **b** cisplatin (25–800 µM) in HBx-HepG2 cells pretreated with 48 h hypoxia (1 % O_2_); **c** flow-cytometry was used to examine the effect of As_2_O_3_ on MMP in HBx-HepG2 cells pretreated with 48 h hypoxia (1 % O_2_); **d** flow-cytometry was used to examine the effect of As_2_O_3_ on MMP in HBx-HepG2 cells pretreated with 48 h hypoxia (1 % O_2_). All data were expressed as mean ± s.e.m., n = 4; ATO = As_2_O_3_; significant differences between hypoxia (1 % O_2_) group and hypoxia (1 % O_2_) + ATO group were indicated as *P < 0.05, **P < 0.01, ***P < 0.001 (unpaired t-test)

Effects of As_2_O_3_ on hypoxia-induced resistance to 5-FU and cisplatin in HBx-HepG2 cells were evaluated by MTT assay. Pretreatment of As_2_O_3_ (1 μM) for 24 h alone did not significantly reduced the cell viability (data not shown). As_2_O_3_ pretreatment for 24 h and subsequent 5-FU (50–1600 µM) or cisplatin (25–800 μM) treatment significantly reduced the cell viability in HBx-HepG2 pre-incubated in hypoxia (1 % O_2_) for 48 h, compared with treatment of 5-FU or cisplatin alone (*P* < 0.05; n = 4, Fig. 5a, b).

### Effect of AsO_3_ on MMP and cell cycle in HBx-HepG2 cells

To further investigate the mechanisms of As_2_O_3_ on the hypoxia-induced chemo-resistance, flow cytometry was used to determine the MMP and cell cycle in HBx-HepG2 cells. Pretreatment of As_2_O_3_ for 24 h significantly increased the MMP in HBx-HepG2 cells pre-incubated in hypoxia (1 % O_2_) for 48 h compared with control (*P* < 0.05; n = 4, Fig. 5c). In addition, pretreatment of As_2_O_3_ for 24 h significantly decreased the percentage of cells in G_0_/G_1_ phase with an associated increase in the percentage of cells in S and G_2_/M phase in HBx-HepG2 cells pre-incubated in hypoxia (1 % O_2_) for 48 h compared with control (*P* < 0.05; n = 4, Fig. 5d).

### Effect of As_2_O_3_ on expression of MDR related genes and proteins

**Fig. 6:**
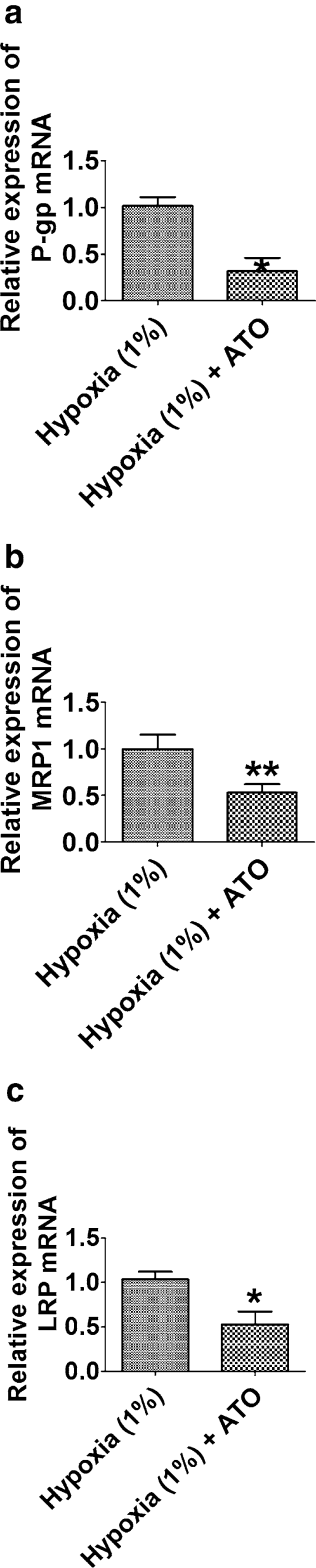
Effect of As_2_O_3_ (1 µM) on the expression levels of P-gp, MRP1 and LRP mRNA inHBx-HepG2 cells pretreated with hypoxia (1 % O_2_). qRT-PCR assay was used to examine the effect of As_2_O_3_ on the expression levels of **a** P-gp, **b** MRP1 and **c** LRP mRNA in HBx-HepG2 cells pretreated with hypoxia (1 % O_2_). All data were expressed as mean ± s.e.m., n = 4; significantly differences between hypoxia (1 % O_2_) group and hypoxia (1 % O_2_) + ATO group were indicated as *P < 0.05, **P < 0.01 (unpaired t-test)

**Fig. 7:**
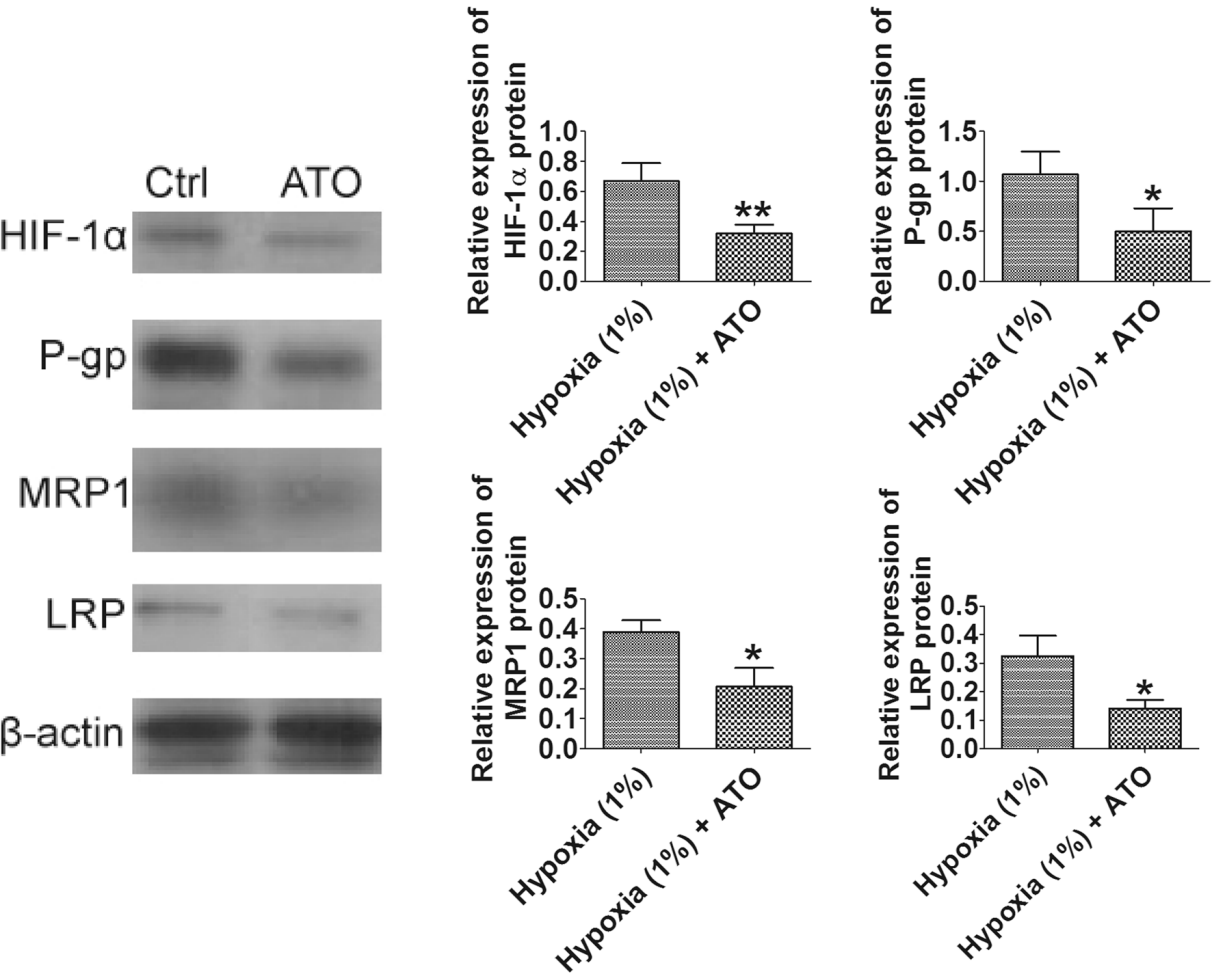
Effect of As_2_O_3_ (1 µM) on the expression levels of P-gp, MRP1 and LRP protein in HBx-HepG2 cells pretreated with hypoxia (1 % O_2_). Western blot assay was used to examine the effect of As_2_O_3_ on the expression levels of P-gp, MRP1 and LRP protein in HBx-HepG2 cells pretreated with hypoxia (1 % O_2_), band densities were normalized to β-actin. All data were expressed as mean ± s.e.m., n = 4; significant differences between hypoxia (1 % O_2_) group and hypoxia (1 % O_2_) + ATO group were indicated as *P < 0.05, **P < 0.01 (unpaired t-test)

To investigate if As_2_O_3_ pretreatment altered the expression of MDR related genes and proteins, qRT-PCR was employed to determine expression levels of P-gp, MRP1 and LRP mRNA, and western blot assay was used to measure the expression levels of HIF-1α, P-gp, MRP1, and LRP proteins. Our qRT-PCR results showed that As_2_O_3_ pretreatment significantly decreased the expression levels of P-gp, MRP1 and LRP mRNA in HBx-HepG2 cells pre-incubated in hypoxia (1 % O_2_) for 48 h when compared with control (*P* < 0.05; n = 4, Fig. 6). Western blot results further confirmed that As_2_O_3_ pretreatment significantly down-regulated the expression of HIF-1α, MRP1, and LRP proteins in HBx-HehpG2 cells pre-incubated in hypoxia (1 % O_2_) for 48 h compared with control (*P* < 0.05; n = 4, Fig. 7).

### Effect of As_2_O_3_ in combination with 5-FU treatment on expression of apoptotic-related factors

**Fig. 8:**
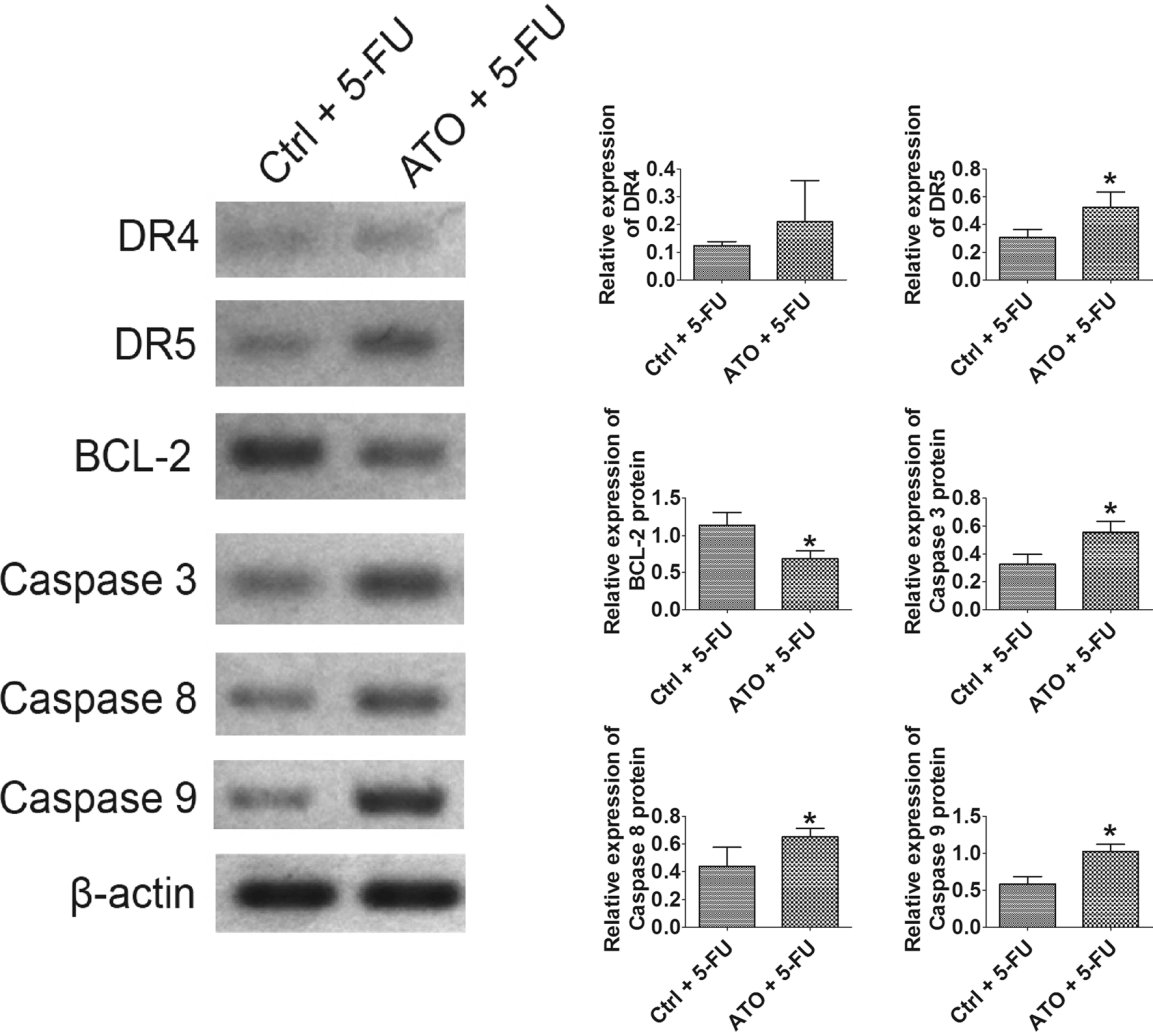
Effect of As_2_O_3_ (1 µM) + 5-FU (400 µM) on the expression levels of apoptotic-related factors protein in HBx-HepG2 cells pretreated with hypoxia (1 % O_2_). Western blot assay was used to examine the effect of As_2_O_3_ + 5-FU on the expression levels of DR4, DR5, BCL-2, caspase 3, caspase 8 and caspase 9 protein in HBx-HepG2 cells pretreated with hypoxia (1 % O_2_), band densities were normalized to β-actin. All data were expressed as mean ± s.e.m., n = 4; significant differences between Ctrl + 5-FU group and ATO + 5-FU group were indicated as *P < 0.05 (unpaired t-test)

To investigate how the apoptotic signaling pathway is affected by As_2_O_3_ in combination with 5-FU treatment, western blot assay was used to determine the protein expression levels of apoptotic-related factors. The combinational treatment significantly increased the expression protein levels of DR5, caspase 3, caspase 8, and caspase 9 (*P* < 0.05; n = 4, Fig. 8), and decreased the protein expression level of BCL-2 (*P* < 0.05; n = 4, Fig. 8); but had no effect on the protein expression level of DR4 (Fig. 8).

## Discussion

In the present study, we for the first time investigated the effect of As_2_O_3_ on the hypoxia-induced chemo-resistance to 5-FU or cisplatin in HBx-HepG2 cells. Generation of stable HepG2 cells expressing HBx was carried out by transfection of HBx plasmid, and the expression of HBx in HepG2 cells after transfection was further confirmed by qRT-pCR and western blot assays. After generation of HBx-HepG2cells, we moved to examine different hypoxic (5 % O_2_, 3 % O_2_, or 1 % O_2_) conditions on the chemo-sensitivity to 5-FU or cisplatin in HBx-HepG2 cells. 5-FU and cisplatin are widely used as first-line and second-line chemotherapy for metastatic HCC [32]. However, the results still remain poor in advanced cases, especially those with metastatic lesions, often dependent on the acquisition of resistance to therapy. The hypoxic environment in the central area of the growing tumor, dependent on the insufficient neovascularization, seems to be in part responsible for this phenomenon. In fact, hypoxic cells are known to be more resistant to ionizing radiation and chemotherapy [33]. Both 5-FU and cisplatin concentration-dependently inhibited the cell viability of HBx-HepG2 cells under normoxia, but under hypoxia particularly at 1 % O_2_, the inhibitory effects of agents were significantly abrogated. Our results showed also that hypoxia (3 % O_2_ and 1 % O_2_) enhanced the arrest of cell cycle at G_0_/G_1_ phase, and previous studies have also demonstrated hypoxia altered the cell cycle at G_0_/G_1_ phase in different types of cancer cells. For instance, hypoxia (1 % O_2_) enhanced the arrest of cell cycle at G0/G1 phase in colorectal cancer cell lines, which showed chemo-resistance to 5-FU and oxaliplatin [34]. Studies using HepG2 cells also found that hypoxia (1 % O_2_) induced chemo-resistance with an associated arrest of cell cycle at G_0_/G_1_ phase [35]. However, the effect of hypoxia on cell arrest of G_0_/G_1_ phase in HBx-HepG2 cells has been not shown yet, and our results may indicate that chemo-resistance to 5-FU and cisplatin in HBx-HepG2 cells was partly dependent on the arrest of the cell cycle at G0/G1 phase, induced by hypoxia, which seemed to prevent these agents from fully exerting their effects on the cell cycle.

Mitochondria have been found to participate in the process of hypoxic responses in different ways and the MMP can reflect the function of mitochondria [36]. Hypoxia could decrease electron-transport rate determining MMP reduction, increased ROS generation, and enhanced NO synthase [37]. In the present study, we demonstrated that hypoxia reduced the MMP in a concentration-dependent manner. Previous studies also showed that hypoxia could reduce the MMP in the human umbilical cord vein endothelial cells [36]. The reduction of MMP has been linked to the upregulation of HIF-1α protein [37]. It was demonstrated in this study that hypoxia significantly increased the HIF-1α protein expression. The upregulation of HIF-1α has been well documented in various studies. Studies also indicate that HIF-1α, as a nuclear transcript factor, facilities the transcription of the MDR related genes and thus accelerates the synthesis of drug transporter proteins including P-gp, MRP1, and LRP, which functions as drug transporter to reduce intracellular drug accumulation [38–40]. Here, we also confirmed that hypoxia increased the expression levels of P-gp, MRP1 and LRP mRNA and protein in HBx-HepG2 cells. It is possible that upregulation of these MDR related genes and proteins is via the HIF-1α pathway. However, further studies may be required to known down the HIF-1α or overexpress HIF-1α to confirm the role of HIF-1α in the regulation of MDR related genes and proteins in HBx-HepG2 cells.

We then tested if As_2_O_3_ pretreatment could re-sensitize the hypoxia-induced chemo-resistance to 5-FU and cisplatin and explored its underlying mechanism in the HBx-HepG2 cells. Our results showed that following pretreatment with As_2_O_3_ at a non-cytotoxic concentration re-sensitize the hypoxia (1 % O_2_)-induced chemo-resistance to 5-FU and cisplatin in HBx-HepG2 cells. As_2_O_3_ pretreatment could also prevent MMP reduction and G_0_/G_1_ arrest induced by hypoxia. Meanwhile, As_2_O_3_ antagonized increase of HIF-1α protein induced by hypoxia, and it also suppresses the increase in expression levels of P-gp, MRP1, and LRP mRNA and proteins. The mechanisms of As_2_O_3_ re-sensitize chemo-resistance has been proposed in several studies. Zhao et al., demonstrated As_2_O_3_ significantly decreased the expression of GST-π and decreased expression of Topo-II, which indicated these MDR-related enzymes may be contributed to the partial reverse of MDR induced by As_2_O_3_ in K563/A02 cells [20]. Another line of evidence suggested that As_2_O_3_ eliminated chemo-resistance in murine B-cell lymphoma via the overexpression of p28 [21]. Recent study also showed that As_2_O_3_ inhibited the expression of Bcl-2 and Bcl-xL, and increased the expression of Bax in HCC cells, which in turn enhances sorafenib-induced apoptosis in HCC [23]. Our results may suggest that As_2_O_3_ re-sensitizes chemo-resistance may involve the mitochondrial function changes and the alteration of HIF-1α in HBx-HepG2 cells, and other pathway may be also involved, which requires further investigation. In addition, we also found that As_2_O_3_ in combination with 5-FU treatment could activate caspase-related apoptotic signaling pathway possibly via the up-regulation of DR5, which may suggest As_2_O_3_ re-sensitizes chemo-resistance at least owing to up-regulation of DR5, caspase 3, caspase 8, and caspase 9, and down-regulation of BCL-2. It is also of great interest to investigate if other signaling pathway is also involved in the future studies.

Cyclin D1 is a key factor for driving the G_1_/S transition of cell cycle, and is overexpressed in HBV-HCC tissues [41, 42]. Studies showed that As_2_O_3_ synergized with sorafenib to down-regulate the expression on of cyclin D1, resulting in cell arrested at G_0_/G_1_ in HCC cells [23], and our results may suggest that As_2_O_3_ re-sensitized hpyoxia chemo-resistance via down-regulation of cyclin D1, which results in cell cycle arrest at G_0_/G_1_ in HBx-HepG2 cells. The role cyclin D1 in As_2_O_3_-induced cycle cell changes may require further investigation.

In conclusion, our results may suggest that As_2_O_3_ re-sensitizes hypoxia-induced chemo-resistance in HBx-HepG2 via complex pathways, and As_2_O_3_ may be a potential agent that given in combination with other anti-drugs for the treatment of HBV related HCC, which is resistant to chemotherapy.

## Abbreviations

5-FU: 5-fluorouracil
APL: acute promyelocytic leukemia
As_2_O_3_: arsenic trioxide
DMEM: Dulbecco’s modified Eagle medium
HBx: hepatitis virus X protein
HBV: hepatitis B virus
HCC: hepatocellular carcinoma
HIF-1α: hypoxia-inducible factor-1α
MDR: multidrug resistance
MRP1: multidrug resistance protein 1
LRP: lung resistance protein
MMP: mitochondrial membrane potential
P-gp: P-glycoprotein
PVDF: polyvinylidene fluoride

## References

[1] Sinn DH, Lee J, Goo J, Kim K, Gwak GY, Paik YH, Choi MS, Lee JH, Koh KC, Yoo BC (2015). Hepatocellular carcinoma risk in chronic hepatitis B virus-infected compensated cirrhosis patients with low viral load.

[2] Liu XY, Tang SH, Wu SL, Luo YH, Cao MR, Zhou HK, Jiang XW, Shu JC, Bie CQ, Huang SM (2015). Epigenetic modulation of insulin-like growth factor-II overexpression by hepatitis B virus X protein in hepatocellular carcinoma. Am J cancer Res.

[3] Yin D, Huang P, Zhuang B, Zhang H, Yan H, Xiao Z, Li W, Zhang J, Tang Q, Hu K (2015). Hepatitis B virus X protein (HBx) is responsible for resistance to targeted therapies in hepatocellular carcinoma: ex vivo culture evidence. Clin Cancer Res.

[4] Koike K (2002). Hepatocarcinogenesis in hepatitis viral infection: lessons from transgenic mouse studies. J Gastroenterol.

[5] Chami M, Ferrari D, Nicotera P, Paterlini-Brechot P, Rizzuto R (2003). Caspase-dependent alterations of Ca^2+^ signaling in the induction of apoptosis by hepatitis B virus X protein. J Biol Chem.

[6] Kim HJ, Kim SY, Kim J, Lee H, Choi M, Kim JK, Ahn JK (2008). Hepatitis B virus X protein induces apoptosis by enhancing translocation of Bax to mitochondria. IUBMB Life.

[7] Kim WH, Hong F, Jaruga B, Zhang ZS, Fan SJ, Liang TJ, Gao B (2005). Hepatitis B virus X protein sensitizes primary mouse hepatocytes to ethanol- and TNF-alpha-induced apoptosis by a caspase-3-dependent mechanism. Cell Mol Immunol.

[8] Petraccia L, Onori P, Sferra R, Lucchetta MC, Liberati G, Grassi M, Gaudio E (2003). MDR (multidrug resistance) in hepatocarcinoma clinical-therapeutic implications. La Clinica Tera.

[9] Rigalli JP, Ciriaci N, Arias A, Ceballos MP, Villanueva SS, Luquita MG, Mottino AD, Ghanem CI, Catania VA, Ruiz ML (2015). Regulation of multidrug resistance proteins by genistein in a hepatocarcinoma cell line: impact on sorafenib cytotoxicity. PLoS One.

[10] Tong SW, Yang YX, Hu HD, An X, Ye F, Hu P, Ren H, Li SL, Zhang DZ (2012). Proteomic investigation of 5-fluorouracil resistance in a human hepatocellular carcinoma cell line. J Cell Biochem.

[11] Semenza GL (2000). Hypoxia, clonal selection, and the role of HIF-1 in tumor progression. Crit Rev Biochem Mol Biol.

[12] Du C, Weng X, Hu W, Lv Z, Xiao H, Ding C, Gyabaah OA, Xie H, Zhou L, Wu J (2015). Hypoxia-inducible MiR-182 promotes angiogenesis by targeting RASA1 in hepatocellular carcinoma. J Exp Clin Cancer Res CR.

[13] Chen MC, Hsu WL, Hwang PA, Chou TC (2015). Low Molecular Weight Fucoidan Inhibits Tumor Angiogenesis through Downregulation of HIF-1/VEGF Signaling under Hypoxia. Marine Drugs.

[14] Denko NC, Fontana LA, Hudson KM, Sutphin PD, Raychaudhuri S, Altman R, Giaccia AJ (2003). Investigating hypoxic tumor physiology through gene expression patterns. Oncogene.

[15] Prabhakar NR, Semenza GL (2012). Adaptive and maladaptive cardiorespiratory responses to continuous and intermittent hypoxia mediated by hypoxia-inducible factors 1 and 2. Physiol Rev.

[16] Shen ZX, Chen GQ, Ni JH, Li XS, Xiong SM, Qiu QY, Zhu J, Tang W, Sun GL, Yang KQ (1997). Use of arsenic trioxide (As_2_O_3_) in the treatment of acute promyelocytic leukemia (APL): II. Clinical efficacy and pharmacokinetics in relapsed patients. Blood.

[17] Chen GQ, Shi XG, Tang W, Xiong SM, Zhu J, Cai X, Han ZG, Ni JH, Shi GY, Jia PM (1997). Use of arsenic trioxide (As_2_O_3_) in the treatment of acute promyelocytic leukemia (APL): I. As_2_O_3_ exerts dose-dependent dual effects on APL cells. Blood.

[18] Carney DA (2008). Arsenic trioxide mechanisms of action–looking beyond acute promyelocytic leukemia. Leuk Lymphoma.

[19] Miller WH, Schipper HM, Lee JS, Singer J, Waxman S (2002). Mechanisms of action of arsenic trioxide. Cancer Res.

[20] Zhao D, Jiang Y, Dong X, Liu Z, Qu B, Zhang Y, Ma N, Han Q (2011). Arsenic trioxide reduces drug resistance to adriamycin in leukemic K562/A02 cells via multiple mechanisms. Biomed Pharmacother.

[21] Giri U, Terry NH, Kala SV, Lieberman MW, Story MD (2005). Elimination of the differential chemoresistance between the murine B-cell lymphoma LY-ar and LY-as cell lines after arsenic (As_2_O_3_) exposure via the overexpression of gsto1 (p28). Cancer Chemother Pharmacol.

[22] Subbarayan PR, Lee K, Ardalan B (2010). Arsenic trioxide suppresses thymidylate synthase in 5-FU-resistant colorectal cancer cell line HT29 In Vitro re-sensitizing cells to 5-FU. Anticancer Res.

[23] Zhai B, Jiang X, He C, Zhao D, Ma L, Xu L, Jiang H, Sun X (2015). Arsenic trioxide potentiates the anti-cancer activities of sorafenib against hepatocellular carcinoma by inhibiting Akt activation. Tumour Biol J Int Soc Oncodevelopmental Biol Med.

[24] Lunghi P, Giuliani N, Mazzera L, Lombardi G, Ricca M, Corradi A, Cantoni AM, Salvatore L, Riccioni R, Costanzo A (2008). Targeting MEK/MAPK signal transduction module potentiates ATO-induced apoptosis in multiple myeloma cells through multiple signaling pathways. Blood.

[25] Szegezdi E, Cahill S, Meyer M, O’Dwyer M, Samali A (2006). TRAIL sensitisation by arsenic trioxide is caspase-8 dependent and involves modulation of death receptor components and Akt. Br J Cancer.

[26] Fayyaz S, Yaylim I, Turan S, Kanwal S, Farooqi AA (2014). Hepatocellular carcinoma: targeting of oncogenic signaling networks in TRAIL resistant cancer cells. Mol Biol Rep.

[27] Farooqi AA, Yaylim I, Ozkan NE, Zaman F, Halim TA, Chang HW (2014). Restoring TRAIL mediated signaling in ovarian cancer cells. Archivum immunologiae et therapiae experimentalis.

[28] Farooqi AA, Fayyaz S, Tahir M, Iqbal MJ, Bhatti S (2012). Breast cancer proteome takes more than two to tango on TRAIL: beat them at their own game. J Membr Biol.

[29] Farooqi AA, Gadaleta CD, Ranieri G, Fayyaz S, Marech I. New frontiers in promoting trail-mediated cell death: focus on natural sensitizers, mirnas, and nanotechnological advancements. Cell Biochem Biophys. 2015;1–8. 10.1007/s12013-015-0712-7 26972296

[30] Wu X, Shi J, Wu Y, Tao Y, Hou J, Meng X, Hu X, Han Y, Jiang W, Tang S (2010). Arsenic trioxide-mediated growth inhibition of myeloma cells is associated with an extrinsic or intrinsic signaling pathway through activation of TRAIL or TRAIL receptor 2. Cancer Biol Ther.

[31] Kim EH, Yoon MJ, Kim SU, Kwon TK, Sohn S, Choi KS (2008). Arsenic trioxide sensitizes human glioma cells, but not normal astrocytes, to TRAIL-induced apoptosis via CCAAT/enhancer-binding protein homologous protein-dependent DR5 up-regulation. Cancer Res.

[32] Song MJ, Bae SH, Chun HJ, Choi JY, Yoon SK, Park JY, Han KH, Kim YS, Yim HJ, Um SH (2015). A randomized study of cisplatin and 5-FU hepatic arterial infusion chemotherapy with or without adriamycin for advanced hepatocellular carcinoma. Cancer Chemother Pharmacol.

[33] Harrison L, Blackwell K (2004). Hypoxia and anemia: factors in decreased sensitivity to radiation therapy and chemotherapy?. Oncologist.

[34] Murono K, Tsuno NH, Kawai K, Sasaki K, Hongo K, Kaneko M, Hiyoshi M, Tada N, Nirei T, Sunami E (2012). SN-38 overcomes chemoresistance of colorectal cancer cells induced by hypoxia, through HIF1alpha. Anticancer Res.

[35] Xu Z, Liu E, Peng C, Li Y, He Z, Zhao C, Niu J (2012). Role of hypoxia-inducible-1alpha in hepatocellular carcinoma cells using a Tet-on inducible system to regulate its expression in vitro. Oncol Rep.

[36] Li MM, Wu LY, Zhao T, Xiong L, Huang X, Liu ZH, Fan XL, Xiao CR, Gao Y, Ma YB (2011). The protective role of 5-HMF against hypoxic injury. Cell Stress Chaperones.

[37] Solaini G, Baracca A, Lenaz G, Sgarbi G (2010). Hypoxia and mitochondrial oxidative metabolism. Biochim Biophys Acta.

[38] Lee JW, Bae SH, Jeong JW, Kim SH, Kim KW (2004). Hypoxia-inducible factor (HIF-1)alpha: its protein stability and biological functions. Exp Mol Med.

[39] Nath B, Szabo G (2012). Hypoxia and hypoxia inducible factors: diverse roles in liver diseases. Hepatology (Baltimore, Md).

[40] Pugh CW, Ratcliffe PJ (2003). Regulation of angiogenesis by hypoxia: role of the HIF system. Nat Med.

[41] Malumbres M, Barbacid M (2009). Cell cycle, CDKs and cancer: a changing paradigm. Nat Rev Cancer.

[42] Parekh P, Rao KV (2007). Overexpression of cyclin D1 is associated with elevated levels of MAP kinases, Akt and Pak1 during diethylnitrosamine-induced progressive liver carcinogenesis. Cell Biol Int.

